# Associated Factors and Survival Outcomes for Breast Conserving Surgery versus Mastectomy among New Zealand Women with Early-Stage Breast Cancer

**DOI:** 10.3390/ijerph18052738

**Published:** 2021-03-08

**Authors:** Mohammad Shoaib Abrahimi, Mark Elwood, Ross Lawrenson, Ian Campbell, Sandar Tin Tin

**Affiliations:** 1 Section of Epidemiology and Biostatistics, School of Population Health, The University of Auckland, Auckland 1142, New Zealand; mark.elwood@auckland.ac.nz (M.E.); s.tintin@auckland.ac.nz (S.T.T.); 2 Department of NIDEA (National Institute of Demographic and Economic Analysis), Waikato Medical Research Centre, The University of Waikato, Hamilton 3240, New Zealand; Ross.Lawrenson@waikatodhb.health.nz; 3 Department of Strategy, Investment and Transformation, Waikato District Health Board, Hamilton 3204, New Zealand; 4 Breast and General Surgeon, Waikato Hospital, Hamilton 3204, New Zealand; Ian.Campbell@waikatodhb.health.nz

**Keywords:** breast conserving therapy, mastectomy, survival, associated factors

## Abstract

This study aimed to investigate type of loco-regional treatment received, associated treatment factors and mortality outcomes in New Zealand women with early-stage breast cancer who were eligible for breast conserving surgery (BCS). This is a retrospective analysis of prospectively collected data from the Auckland and Waikato Breast Cancer Registers and involves 6972 women who were diagnosed with early-stage primary breast cancer (I-IIIa) between 1 January 2000 and 31 July 2015, were eligible for BCS and had received one of four loco-regional treatments: breast conserving surgery (BCS), BCS followed by radiotherapy (BCS + RT), mastectomy (MTX) or MTX followed by radiotherapy (MTX + RT), as their primary cancer treatment. About 66.1% of women received BCS + RT, 8.4% received BCS only, 21.6% received MTX alone and 3.9% received MTX + RT. Logistic regression analysis was used to identify demographic and clinical factors associated with the receipt of the BCS + RT (standard treatment). Differences in the uptake of BCS + RT were present across patient demographic and clinical factors. BCS + RT was less likely amongst patients who were older (75+ years old), were of Asian ethnicity, resided in impoverished areas or areas within the Auckland region and were treated in a public healthcare facility. Additionally, BCS + RT was less likely among patients diagnosed symptomatically, diagnosed during 2000–2004, had an unknown tumour grade, negative/unknown oestrogen and progesterone receptor status or tumour sizes ≥ 20 mm, ≤50 mm and had nodal involvement. Competing risk regression analysis was undertaken to estimate the breast cancer-specific mortality associated with each of the four loco-regional treatments received. Over a median follow-up of 8.8 years, women who received MTX alone had a higher risk of breast cancer-specific mortality (adjusted hazard ratio: 1.38, 95% confidence interval (CI): 1.05–1.82) compared to women who received BCS + RT. MTX + RT and BCS alone did not have any statistically different risk of mortality when compared to BCS + RT. Further inquiry is needed as to any advantages BCS + RT may have over MTX alternatives.

## 1. Introduction

Breast cancer poses a serious public health issue globally. Worldwide, an estimated 2,088,849 women were diagnosed (46.3/100,000—age standardised rate (*)) and 626,697 deaths (13.0/100,000*) were estimated in 2018 [1]. Comparatively, New Zealand has one of the highest incidences of breast cancer (3504 women diagnosed in 2018, (92.6/100,000*)) yet one of the lowest worldwide mortality (632 deaths in 2018, (10.9/100,000*)) [1].

Surgery is the mainstay treatment for women with early-stage breast cancer. In New Zealand [2], and similarly in other countries [3,4,5], women with stage I–IIIa breast cancer are offered the choice of breast conserving surgery plus radiotherapy (BCS + RT) or mastectomy (MTX). These recommendations were partly based on earlier randomised controlled trials (RCTs) illustrating equivalent survival outcomes between BCS + RT and MTX [6,7]. However, more recent observational studies suggest BCS + RT may offer greater survival advantages compared to MTX [8,9].

A woman’s choice to receive a particular treatment may be influenced by access barriers and inconvenience, such as travel distance to treatment centres, as well as the quantity, duration and costs involved in receiving a particular treatment. Previous research has found factors such as education level, area deprivation, tumour stage, age and ethnicity to be associated with differences in the uptake of loco-regional treatment of breast cancer patients. More specifically, residing in impoverished areas, having a later tumour stage, being older (greater than 49 years) and of Asian/Pacific ethnicity have been associated with a decreased likelihood in BCS + RT uptake [10,11].

This study aims to investigate type of loco-regional treatment received by New Zealand women with early-stage breast cancer, factors associated with the receipt of BCS + RT (standard treatment) and compares breast cancer-specific mortality across BCS, BCS + RT, MTX and MTX + RT surgical treatments.

## 2. Materials and Methods

### 2.1. Study Population

**Figure 1:**
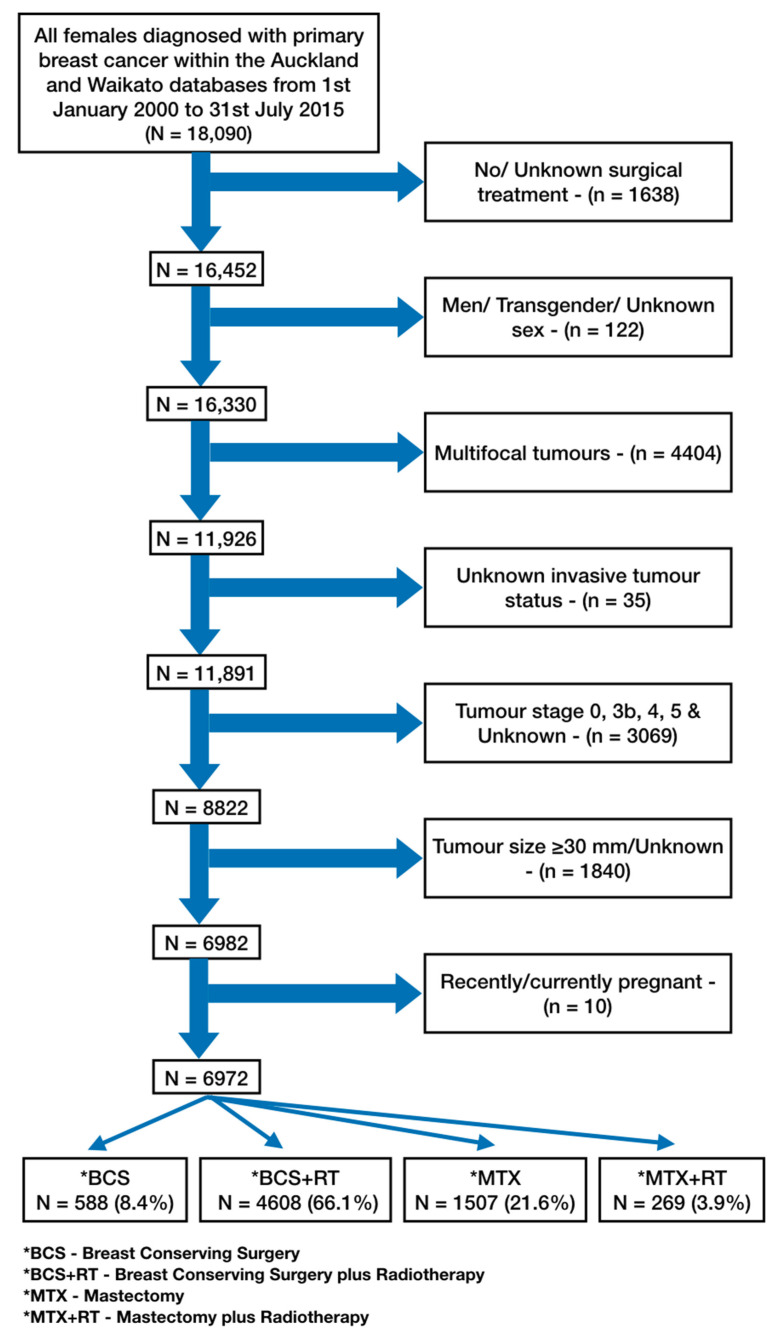
Sample restriction flowchart.

Our sample consisted of 6972 women diagnosed with early-stage (stage I–IIIA) primary breast cancer between 1 January 2000 and 31 July 2015 who were eligible for BCS and underwent one of the four loco-regional treatments: BCS, BCS + RT, MTX or MTX + RT (Figure 1). Women were identified from the Auckland and Waikato breasts cancer registers which encompass four health regions (Waitemata, Auckland, Counties Manukau and Waikato district health boards) with over 2 million residents.

### 2.2. Data Sources

The Auckland and Waikato breast cancer registers are prospectively maintained, opt-out, population-based registers with detailed demographic and clinical data [12]. Information on women identified with breast cancer are extracted in a structured format by trained data entry personnel from all clinical and pathological reports [13]. Record data is extracted from clinical reports, operations records, multi-disciplinary meetings records, oncology reports, palliative care records as well as private and public hospital records [13]. Extracted data is entered into both Auckland and Waikato registers depending on the patient’s area of residence and is cross referenced with the New Zealand Cancer Register (NZCR) and Mortality Collection annually. Under the Cancer Registry Act 1993, all malignant tumours first diagnosed in New Zealand are legally required to be registered in the NZCR, with the exception of basal cell and squamous cell skin tumours [14]. Additionally, all deaths are legally required to be registered in the Mortality Collection [15]. Both the NZCR and Mortality Collection are run and organised by the Ministry of Health [13]. Since 2000, the Auckland and Waikato registers have enrolled virtually all newly diagnosed breast cancer cases within their respective health regions. The Waikato breast cancer register was found to be 99 percent complete when cross referenced with the National Cancer Registry, and the Auckland breast cancer register only had a one percent loss to follow-up [13,16,17].

Ethical approval for the use of anonymised patient data in this study was obtained from the University of Auckland Human Participants Ethics Committee (Ref. No. 21851).

### 2.3. Variables of Interest

The main exposure of interest is the type of loco-regional treatment received after diagnosis of breast cancer, i.e., BCS, BCS + RT, MTX or MTX + RT.

Potential confounders were identified from previous studies as well as the possible confounding effect on the exposure (treatment)–outcome (breast cancer-specific mortality) association [18,19]. Patient demographic factors included age, ethnicity, New Zealand deprivation Index 2013, urban/rural residence, region of residence and whether treatment was undertaken in a public or private facility. Clinical variables included were year of diagnosis, mode of detection (screen-detected or symptomatic), tumour stage, grade, hormone receptor status, histological type, tumour size, lymph node involvement and lympho-vascular invasion (LVI).

The New Zealand deprivation index 2013 is a unique measure of deprivation used in New Zealand to assess the level of deprivation present in a particular area. Here, the New Zealand deprivation score represents the likely deprivation of a given patient based on the area they reside in. The index provides a score out of ten, with one representing the least deprived area and ten representing the most deprived area. The New Zealand deprivation index 2013 is based on nine variables collected at the time of New Zealand’s 2013 census [20].

### 2.4. Statistical Analysis

Logistic regression models were used to assess factors associated with the receipt of BCS + RT (Table 1). Cox models were used in the competing risk analyses to estimate the probability of breast cancer-specific mortality, with breast cancer-specific death being the main failure event of interest and death from other causes being the competing event. Survival time was calculated from date of cancer diagnosis until failure (breast cancer-specific death), a competing event or censorship (Table 2 and Table 3) [21,22]. Patients were censored on the 31 July 2018 if they did not experience failure or a competing event.

Logistic regression analyses controlled for demographic and clinicopathological factors with exception to the competing risk analyses, which also controlled for systemic treatment factors. All statistical analyses were carried out using STATA MP version 16.0 (StataCorp, College Station, TX, USA).

## 3. Results

### 3.1. Patient Characteristics

Of the 6972 women included in this analysis, 588 (8.4%) received BCS alone, 4608 (66.1%) received BCS + RT, 1507 (21.6%) received MTX alone and 269 (3.9%) received MTX + RT. A total of 320 breast cancer-specific deaths occurred: BCS—n = 23 (7.2%), BCS + RT—n = 170 (53.1%), MTX—n = 98 (30.6%) and MTX + RT—n = 29 (9.1%). The median age was 58 (IQR: 50–66) years for the whole sample, 58 (IQR: 49–68) years for those who received BCS only, 58 (IQR: 50–65) years for those who received BCS + RT, 61 (IQR: 51–71) years for those who received MTX alone and 53 (IQR: 45–63) years for those who received MTX + RT (Table 2). Table 2 shows the distribution of demographic, clinical and systemic treatment variables within our sample population across BCS, BCS + RT, MTX and MTX + RT surgical treatment groups. The majority of women in our sample were: aged between 45 and 59, European, resided in urban areas, resided within the Auckland region and treated publicly. Clinically, women in our study were diagnosed between 2010 and 2015, and were most likely to have tumours of stage IA, grade 2, be oestrogen receptor- (ER) and progesterone receptor (PR)-positive, ductal tumours, <20 mm in size, with no nodal involvement, no lymphovascular invasion and likely to receive only hormone therapy.

### 3.2. Factors Associated with the Receipt of BCS + RT

Demographic factors associated with the receipt of BCS + RT were age, ethnicity, deprivation, public/private facility type and region. Older women (75+ years old) were less likely (odds ratio (OR): 0.34, 95% confidence interval (CI): 0.27–0.43) to receive BCS + RT compared to their younger counterparts (<45 years old). Relative to European women, Asian women were less likely to receive BCS + RT (OR: 0.58, 95% CI: 0.48–0.69). Patients residing in deprived areas (deprivation levels 9–10) were less likely to receive BCS + RT (OR: 0.76, 95% CI: 0.64–0.91) compared to those residing in more affluent areas (deprivation level 1–2). Similarly, patients treated through the public system (OR: 0.79, 95% CI: 0.70–0.89) were less likely to receive BCS + RT compared to their privately treated counterparts. Lastly, patients treated in Waikato were more likely to receive BCS + RT (OR: 1.97, 95% CI: 1.70–2.29) compared to Auckland patients.

Clinical factors associated with the receipt of BCS + RT included: year of diagnosis, detection method, hormone receptor status, tumour size and lymph node involvement. Compared to women diagnosed during the 2000–2004 period, those diagnosed between 2005–2009 (OR: 1.24, 95% CI: 1.08–1.42) and 2010–2015 (OR: 1.18, 95% CI: 1.03–1.35) periods were more likely to receive BCS + RT. Compared to women detected through screening, symptomatic women were less likely to receive BCS + RT (OR: 0.60, 95% CI: 0.54–0.68). Patients who were oestrogen (ER) and progesterone (PR) receptor-negative (OR: 0.68, 95% CI: 0.57–0.81) or had an unknown status (OR:0.53, 95% CI:0.36–0.78) were less likely to receive BCS + RT compared to their ER- and PR-positive receptor counterparts. Women with a tumour size ≥ 2–≤50 mm were less likely (OR: 0.63, 95% CI: 0.56–0.71) to receive BCS + RT compared to women with tumour(s) < 20 mm. Lastly, the likelihood of BCS + RT decreased as lymph node involvement increased (0 positive nodes OR: 1.00; 1–3 positive nodes OR: 0.83, 95% CI: 0.72–0.95; 4+ positive nodes OR: 0.60, 95% CI: 0.44–0.80).

### 3.3. Breast Cancer-Specific Death

**Figure 2:**
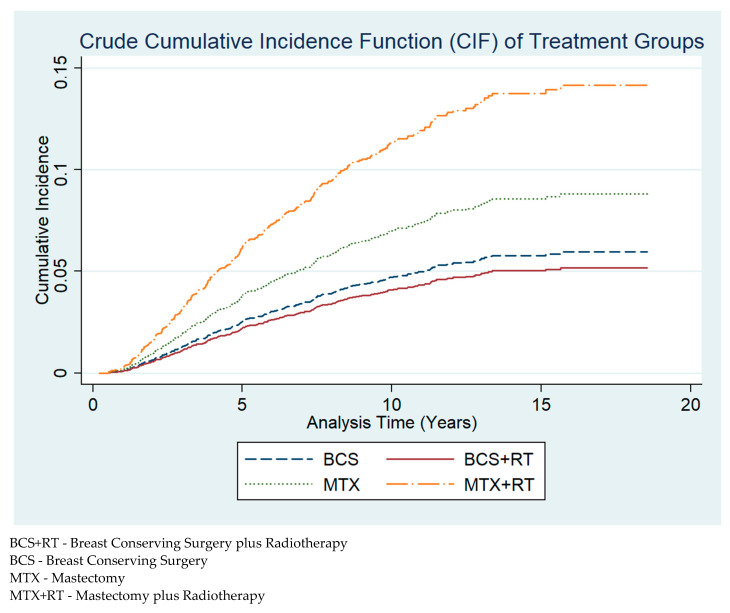
Crude cumulative incidence function (CIF) plot of breast cancer-specific mortality across BCS, BCS + RT, MTX and MTX + RT surgical treatment groups.

There were 320 (4.6%) deaths from breast cancer over a median follow-up period of 8.8 years. The crude cumulative incidence function (CIF) graph (Figure 2) demonstrates that breast cancer-specific mortality risk was highest in women who received MTX + RT, followed by MTX, which has roughly half the risk as that of MTX + RT, and subsequently BCS, closely followed by BCS + RT. After adjustment for demographic, clinical and systemic treatment factors (Table 3), MTX had a higher risk of breast cancer-specific mortality (HR ^3^: 1.38, 95% CI: 1.05–1.82) relative to BCS + RT (Table 3).

## 4. Discussion

### 4.1. Main Findings

In this population-based study involving over 6900 women with early-stage breast cancer, BCS + RT was the most commonly used loco-regional treatment, but its uptake differed significantly by a number of demographic and clinicopathological factors. Compared to women who received BCS + RT, those who received MTX had a higher risk of breast cancer-specific mortality over the median follow-up period of 8.8 years. Patients receiving MTX + RT or BCS alone did not have any statistically different risk of mortality when compared to BCS + RT.

### 4.2. Interpretation

Amongst our cohort of New Zealand women with early-stage (stage I–IIIA) breast cancer, a number of patterns emerged. Those aged 75+ were less likely to receive BCS + RT compared to younger women aged < 45. Younger women may opt for the least invasive of the two surgical procedures due to self-image concerns, whereas elderly women are less likely to be as concerned and opt for the most convenient option [23].

Women residing in more deprived areas were less likely to receive BCS + RT compared to those living in more affluent areas. Deprived neighbourhoods tend to be less likely equipped with radiotherapy treatment facilities and hence women residing in low-deprivation areas would likely face a greater travel distance [24,25,26], cost and inconvenience in receiving BCS + RT treatment compared to women residing in more affluent neighbourhoods [25,27].

Patients residing within the Auckland region were less likely to receive BCS + RT compared to their counterparts in Waikato. Given that Auckland is a more urban area than Waikato and there are less access barriers in urban areas, differences in surgical treatments here may be due to physician influence between the two regions. It could be likely that physicians practising within the Waikato region are more comfortable utilising breast conserving surgical techniques as opposed to mastectomy surgeries and hence their preferences/skills may be indicative of the patient’s final choice. Furthermore, areas with a low ratio of medical oncologists to patients have been shown to result in a greater uptake of BCS + RT, which may be the case in Waikato when compared to Auckland [28].

Patients treated in a public facility were less likely to receive BCS + RT compared to their privately treated counterparts. Private health facilities likely have more resources available relative to demand, translating to less wait times for elective surgeries/treatment [29]. Compared to MTX alone, BCS + RT is more resource-intensive, requiring a multi-faceted team approach and numerous subsequent treatments. Churilla and colleagues have found the likelihood of BCS + RT decreases with a decrease in the density of radiation oncologists present [30]. Thus, the lack of resources and delay in accessing breast cancer treatment within the public sectors could explain the decreased likelihood of BCS + RT uptake relative to privately treated patients.

The likelihood of BCS + RT decreased if patients were diagnosed during 2000–2004, detected symptomatically, had tumour sizes ≥ 20–≤50 mm, negative/unknown oestrogen and progesterone receptor status, unknown grade and with increasing lymph node involvement.

Patients receiving BCS + RT had the lowest risk of breast cancer-specific mortality over a median follow-up period of 8.8 years, with patients treated with MTX alone having a 38% (95% CI: 5–82%) increased risk of mortality comparatively. One reason for these differences may be due to the administration of radiotherapy in BCS, which helps further suppress/kill smaller cancerous cells that may have been missed during surgery, whereas for patients undergoing MTX alone, they rely solely on tumorous growth being detected by their surgeon. Overall, our results are consistent with recent observational studies [8,9,18,31,32,33].

### 4.3. Strengths

To our knowledge, this is the first observational study in New Zealand that identified factors associated with differences in the uptake of surgical treatment for women with early-stage breast cancer and investigated differences in breast cancer mortality across four surgical treatment groups (BCS, BCS + RT, MTX and MTX + RT). We used two opt-out prospectively maintained population-based databases which contained comprehensive and near complete data with respect to patients diagnosed with primary breast cancer [12,13,17]. Linkage to national databases also allowed for the extraction of data pertaining to cause of death and patient comorbidities with minimal loss to follow-up [13,17]. We undertook a competing risks analysis which aims to more accurately reflect risk of breast cancer-specific mortality and has not been undertaken in previous observational studies [19].

### 4.4. Limitations

Despite efforts to control for confounding, residual confounding may still be present as some key variables that could influence choice of surgery and/or mortality were not controlled for due to lack of data availability, e.g., antigen Ki-67 [34], body mass index (BMI), alcohol intake [35], co-morbidities and presence of Breast Cancer Gene 1 (BRCA1) and Breast Cancer Gene 2 (BRCA2) gene mutations. Lack of routine Human Epidermal Growth Factor Receptor 2 (HER2) testing prior to 2006 in our database resulted in a significant number of patients with an unknown HER2 status (n = 2147—20.9% unknown). Socioeconomic status (NZDep2013) was based on area level deprivation rather than individual level deprivation.

### 4.5. Implications

Demographic differences in loco-regional treatment of women with early-stage breast cancer highlight some of the largely modifiable inequities currently present in our healthcare system, providing room for gaining more equitable outcomes. Our findings underscore more efforts to identify and alleviate the access barriers the disadvantaged populations are faced with.

BCS + RT has certain advantages over MTX alone. BCS + RT is a minimally invasive treatment with reduced post-operative complications, a faster recovery time and patients generally report a better overall self-image [23,36]. Where appropriate, BCS + RT should be recommended rather than MTX. It is important to note that MTX should still remain the treatment of choice for certain groups of women, such as those who have a contraindication to radiation therapy, e.g., women with certain connective tissue disorders, pregnancy, certain hereditary gene mutations such as BRCA1 and BRCA2 or where large tumour to breast ratio makes BCS + RT impractical [2,37].

Currently, in New Zealand, women with early-stage breast cancer are treated based on the guidelines set out by the New Zealand Guidelines Group [2]. The option between BCS + RT and MTX exists for women [2], implying survival outcomes for both treatments are the same if not very similar. It is worth inquiring further as to the survival advantages of BCS + RT and MTX treatments given the findings from this study and a growing body of international population-based literature.

## 5. Conclusions

Overall, our study shows that older women, Asians, those residing in impoverished areas or in the Auckland region and those who were diagnosed with symptomatic cancer were less likely to receive BCS + RT even after taking clinical factors into account. We also found that compared to women who received BCS + RT, women who received MTX alone had a greater risk of breast cancer-specific mortality. Our findings underscore the need for more efforts needed to ensure equitable cancer care in New Zealand.

## Figures and Tables

**Table 1:** Odds ratios (OR) from logistic regression across BCS + RT ^1^ vs. BCS ^2^/MTX ^3^/MTX + RT ^4^.

Covariates	Crude OR	Adjusted OR
DEMOGRAPHIC VARIABLES		
**Age**		
<45	1.00	1.00
45–59	1.59 (1.35–1.87)	1.14 (0.96–1.36)
60–74	1.52 (1.28–1.79)	1.01 (0.84–1.22)
75+	0.40 (0.33–0.50)	0.34 (0.27–0.43)
**Ethnicity**		
European	1.00	1.00
Maori	0.91 (0.77–1.08)	0.91 (0.75–1.10)
Pacific	0.79 (0.60–1.03)	0.94 (0.70–1.27)
Asian	0.58 (0.49–0.69)	0.58 (0.48–0.69)
Other	1.12 (0.76–0.65)	1.02 (0.68–1.53)
Unknown	1.06 (0.78–1.44)	1.02 (0.74–1.41)
**NZ Deprivation**		
1–2	1.00	1.00
3–4	1.09 (0.93–1.27)	1.16 (0.99–1.37)
5–6	0.99 (0.85–1.16)	1.00 (0.85–1.18)
7–8	1.06 (0.90–1.25)	1.04 (0.87–1.25)
9–10	0.80 (0.69–0.93)	0.76 (0.64–0.91)
Unknown	0.91 (0.68–1.22)	1.10 (0.38–3.20)
**Urban Rural**		
Urban	1.00	1.00
Rural	1.28 (1.05–1.57)	0.97 (0.78–1.20)
Unknown	0.92 (0.70–1.21)	0.94 (0.33–2.70)
**Region**		
Auckland	1.00	1.00
Waikato	1.46 (1.29–1.65)	1.97 (1.70–2.29)
**Public/Private Treatment**		
Private	1.00	1.00
Public	0.76 (0.69–0.85)	0.79 (0.70–0.89)
CLINICOPATHOLOGICAL VARIABLES		
**Year of Diagnosis**		
2000–2004	1.00	1.00
2005–2009	1.26 (1.11–1.44)	1.24 (1.08–1.42)
2010–2015	1.21 (1.07–1.37)	1.18 (1.03–1.35)
**Screened/Symptomatic**		
Screened	1.00	1.00
Symptomatic	0.47 (0.42–0.52)	0.60 (0.54–0.68)
**Grade**		
1	1.00	1.00
2	0.71 (0.63–0.80)	0.90 (0.78–1.02)
3	0.65 (0.56–0.74)	1.09 (0.91–1.31)
Unknown	0.37 (0.21–0.63)	0.45 (0.25–0.81)
**Hormone Receptor Status**		
ER ^5^- and PR ^6^-positive	1.00	1.00
ER ^5^- and PR ^6^-negative	1.49 (1.30–1.71)	0.68 (0.57–0.81)
ER ^5^- or PR ^6^-positive	1.03 (1.08–1.55)	0.89 (0.76–1.03)
Unknown	0.89 (0.62–1.28)	0.53 (0.36–0.78)
**Histology**		
Ductal	1.00	1.00
Lobular	0.89 (0.75–1.07)	0.89 (0.73–1.09)
Others	1.06 (0.87–1.29)	1.04 (0.84–1.29)
**Tumour Size**		
<20 mm	1.00	1.00
≥20–≤50 mm	0.48 (0.43–0.54)	0.63 (0.56–0.71)
		
**Positive Lymph Node Status**		
0	1.00	1.00
1–3	0.71 (0.62–0.80)	0.83 (0.72–0.95)
4+	0.47 (0.36–0.61)	0.60 (0.44–0.80)
**Lymphovascular Invasion**		
No	1.00	1.00
Yes	0.79 (0.70–0.90)	1.01 (0.87–1.17)

^1^ Breast Conserving Surgery plus Radiotherapy (BCS + RT); ^2^ Breast Conserving Surgery (BCS); ^3^ Mastectomy (MTX); ^4^ Mastectomy plus Radiotherapy (MTX + RT); ^5^ Oestrogen Receptor (ER); ^6^ Progesterone Receptor (PR).

**Table 2:** Baseline characteristics of surgical treatment groups by demographic, clinical and systemic treatment variables (See Appendix A, Table A1 for table with row percentages).

Factors	Total	BCS ^1^		BCS + RT ^2^		MTX ^3^		MTX + RT ^4^	
		No.	Col%	No.	Col%	No.	Col%	No.	Col%
**Total No.**	6972	588	100.0	4608	100.0	1507	100.0	269	100.0
DEMOGRAPHIC VARIABLES									
**Median Age**	58 (50–66) ^5^	58 (49–68) ^5^		58 (50–65) ^5^		61 (51–71) ^5^		53 (45–63) ^5^	
**Age Group**									
<45	789	78	13.3	475	10.3	172	11.4	64	23.8
45–59	3006	236	40.1	2124	46.1	531	35.2	115	42.8
60–74	2538	184	31.3	1767	38.3	523	34.7	64	23.8
75+	639	90	15.3	242	5.3	281	18.6	26	9.7
**Ethnicity**									
European	5180	429	73.0	3495	75.8	1063	70.5	193	71.7
Māori	653	50	8.5	427	9.3	139	9.2	37	13.8
Pacific	229	29	4.9	142	3.1	53	3.5	5	1.9
Other	715	58	9.9	410	8.9	218	14.5	29	10.8
Unknown	195	22	3.7	134	2.9	34	2.3	5	1.9
**NZ Deprivation**									
1–2	1534	140	23.8	1021	22.2	307	20.4	66	24.5
3–4	1394	108	18.4	954	20.7	296	19.6	36	13.4
5–6	1415	118	20.1	940	20.4	298	19.8	59	21.9
7–8	1121	80	13.6	760	16.5	233	15.5	48	17.8
9–10	1289	117	19.9	792	17.2	326	21.6	54	20.1
Unknown	219	25	4.3	141	3.1	47	3.1	6	2.2
**Main Urban Areas**									
Urban	6252	515	87.6	4112	89.2	1377	91.4	248	92.2
Rural	496	46	7.8	353	7.7	81	5.4	16	5.9
Unknown	224	27	4.6	143	3.1	49	3.3	5	1.9
**Region**									
Auckland	5479	468	79.6	3526	76.5	1287	85.4	198	73.6
Waikato	1493	120	20.4	1082	23.5	220	14.6	71	26.4
**Public/Private**									
Public	2245	170	28.9	1574	34.2	416	27.6	85	31.6
Private	4727	418	71.1	3034	65.8	1091	72.4	184	68.4
CLINICAL VARIABLES									
**Year of Diagnosis**									
2000–2004	1861	143	24.3	1165	25.3	456	30.3	97	36.1
2005–2009	2240	148	25.2	1520	33.0	489	32.4	83	30.9
2010–2015	2871	297	50.5	1923	41.7	562	37.3	89	33.1
**Screen-Detected/Symptomatic**									
Screen-Detected	3605	283	48.1	2675	58.1	587	39.0	60	22.3
Symptomatic	3367	305	51.9	1933	41.9	920	61.0	209	77.7
**Stage**									
IA	4521	434	73.8	3192	69.3	854	56.7	41	15.2
IB	193	17	2.9	133	2.9	37	2.5	6	2.2
IIA	1587	104	17.7	963	20.9	452	30.0	68	25.3
IIB	446	27	4.6	208	4.5	149	9.9	62	23.0
IIIA	225	6	1.0	112	2.4	15	1.0	92	34.2
**Grade**									
1	2191	218	37.1	1572	34.1	373	24.8	28	10.4
2	3182	251	42.7	2050	44.5	756	50.2	125	46.5
3	1545	109	18.5	960	20.8	362	24.0	114	42.4
Unknown	54	10	1.7	26	0.6	16	1.1	2	0.7
**Hormone Receptor Status**									
ER ^6^ and PR ^7^ negative	1018	72	12.2	600	13.0	264	17.5	82	30.5
ER ^6^ and PR ^7^ positive	4801	413	70.2	3270	71.0	975	64.7	143	53.2
ER ^6^ or PR ^7^ positive	1016	90	15.3	661	14.3	227	15.1	38	14.1
Unknown	137	13	2.2	77	1.7	41	2.7	6	2.2
**Histology**									
Ductal	5909	472	80.3	3912	84.9	1286	85.3	239	88.8
Lobular	567	45	7.7	361	7.8	140	9.3	21	7.8
Other	496	71	12.1	335	7.3	81	5.4	9	3.3
**Tumour Size (mm)**									
<20	4847	445	75.7	3452	74.9	859	57.0	91	33.8
≥20–≤50	2125	143	24.3	1156	25.1	648	43.0	178	66.2
**Positive Lymph Node**									
0	5453	498	84.7	3717	80.7	1169	77.6	69	25.7
1–3	1295	85	14.5	779	16.9	323	21.4	108	40.1
4+	224	5	0.9	112	2.4	15	1.0	92	34.2
**Lympho-vascular Invasion**									
Yes	5799	508	86.4	3885	84.3	1254	83.2	152	56.5
No	1173	80	13.6	723	15.7	253	16.8	117	43.5
SYSTEMIC TREATMENT VARIABLES									
**Systemic Treatment**									
None	2303	275	46.8	1464	31.8	545	36.2	19	7.1
Both	932	44	7.5	602	13.1	180	11.9	106	39.4
Chemotherapy	711	47	8.0	449	9.7	141	9.4	74	27.5
Hormonal therapy	2961	204	34.7	2064	44.8	624	41.4	69	25.7
Unknown	65	18	3.1	29	0.6	17	1.1	1	0.4

^1^ Breast Conserving Surgery (BCS); ^2^ Breast Conserving Surgery plus Radiotherapy (BCS + RT); ^3^ Mastectomy (MTX); ^4^ Mastectomy plus Radiotherapy (MTX + RT); ^5^ Interquartile Range^; 6^ Oestrogen Receptor (ER); ^7^ Progesterone Receptor (PR).

**Table 3:** Hazard ratios (HR) for breast cancer-specific mortality from competing risks analysis.

Type of Loco-Regional Treatment	Crude HR (95% CI)	Adjusted HR (95% CI) ^1^	Adjusted HR (95% CI) ^2^	Adjusted HR (95% CI) ^3^
BCS + RT ^4^	1.00	1.00	1.00	1.00
BCS ^5^	1.16 (0.75–1.78)	1.13 (0.73–1.76)	1.21 (0.77–1.88)	1.11 (0.71–1.75)
MTX ^6^	1.73 (1.35–2.22)	1.78 (1.37–2.30)	1.45 (1.10–1.90)	1.38 1.05–1.82)
MTX + RT ^7^	2.87 (1.93–4.27)	2.73 (1.82–4.09)	1.02 (0.65–1.60)	1.05 (0.66–1.67)

^1^ Adjusted for demographic factors: age, ethnicity, NZ deprivation, urban status, region, public/private; ^2^ Adjusted as above and for clinic-pathological factors: screen-detected/symptomatic, grade, hormone receptor status, histology, tumour size, lymph node status, lympho-vascular invasion (LVI); ^3^ Adjusted as above and for systemic treatment factors: chemo and hormonal therapies; ^4^ Breast Conserving Surgery plus Radiotherapy (BCS + RT); ^5^ Breast Conserving Surgery (BCS); ^6^ Mastectomy (MTX); ^7^ Mastectomy plus Radiotherapy (MTX + RT).

## Data Availability

No new data were created or analysed in this study. Ethical approval is restricted to the use of the data by the named investigators. Access by others will require application to the cancer registers used and to the ethical committees.

## Appendix A

**Table A1:** Baseline tables by row percentage.

Factors	Total	BCS ^1^		BCS + RT ^2^		MTX ^3^		MTX + RT ^4^	
		No.	Row%	No.	Row%	No.	Row%	No.	Row%
**Total No.**	6972	588	8.4	4608	66.1	1507	21.6	269	3.9
DEMOGRAPHIC VARIABLES									
**Median Age**	58 (50–66) ^5^	58 (49–68) ^5^		58 (50–65) ^5^		61 (51–71) ^5^		53 (45–63) ^5^	
**Age Group**									
<45	789	78	9.9	475	60.2	172	21.8	64	8.1
45–59	3006	236	7.9	2124	70.7	531	17.7	115	3.8
60–74	2538	184	7.2	1767	69.6	523	20.6	64	2.5
75+	639	90	14.1	242	37.9	281	44.0	26	4.1
**Ethnicity**									
European	5180	429	8.3	3495	67.5	1063	20.5	193	3.7
Māori	653	50	7.7	427	65.4	139	21.3	37	5.7
Pacific	229	29	12.7	142	62.0	53	23.1	5	2.2
Other	715	58	8.1	410	57.3	218	30.5	29	4.1
Unknown	195	22	11.3	134	68.7	34	17.4	5	2.6
**NZ Deprivation**									
1–2	1534	140	9.1	1021	66.6	307	20.0	66	4.3
3–4	1394	108	7.7	954	68.4	296	21.2	36	2.6
5–6	1415	118	8.3	940	66.4	298	21.1	59	4.2
7–8	1121	80	7.1	760	67.8	233	20.8	48	4.3
9–10	1289	117	9.1	792	61.4	326	25.3	54	4.2
Unknown	219	25	11.4	141	64.4	47	21.5	6	2.7
**Main Urban Areas**									
Urban	6252	515	8.2	4112	65.8	1377	22.0	248	4.0
Rural	496	46	9.3	353	71.2	81	16.3	16	3.2
Unknown	224	27	12.1	143	63.8	49	21.9	5	2.2
**Region**									
Auckland	5479	468	8.5	3526	64.4	1287	23.5	198	3.6
Waikato	1493	120	8.0	1082	72.5	220	14.7	71	4.8
**Public/Private**									
Public	2245	170	7.6	1574	70.1	416	18.5	85	3.8
Private	4727	418	8.8	3034	64.2	1091	23.1	184	3.9
CLINICAL VARIABLES									
**Year of Diagnosis**									
2000–2004	1861	143	7.7	1165	62.6	456	24.5	97	5.2
2005–2009	2240	148	6.6	1520	67.9	489	21.8	83	3.7
2010–2015	2871	297	10.3	1923	67.0	562	19.6	89	3.1
**Screen-Detected/Symptomatic**									
Screen-Detected	3605	283	7.9	2675	74.2	587	16.3	60	1.7
Symptomatic	3367	305	9.1	1933	57.4	920	27.3	209	6.2
**Stage**									
IA	4521	434	9.6	3192	70.6	854	18.9	41	0.9
IB	193	17	8.8	133	68.9	37	19.2	6	3.1
IIA	1587	104	6.6	963	60.7	452	28.5	68	4.3
IIB	446	27	6.1	208	46.6	149	33.4	62	13.9
IIIA	225	6	2.7	112	49.8	15	6.7	92	40.9
**Grade**									
1	2191	218	9.9	1572	71.7	373	17.0	28	1.3
2	3182	251	7.9	2050	64.4	756	23.8	125	3.9
3	1545	109	7.1	960	62.1	362	23.4	114	7.4
Unknown	54	10	18.5	26	48.1	16	29.6	2	3.7
**Hormone Receptor Status**									
ER ^6^ and PR ^7^ negative	1018	72	7.1	600	58.9	264	25.9	82	8.1
ER ^6^ and PR ^7^ positive	4801	413	8.6	3270	68.1	975	20.3	143	3.0
ER ^6^ or PR ^7^ positive	1016	90	8.9	661	65.1	227	22.3	38	3.7
Unknown	137	13	9.5	77	56.2	41	29.9	6	4.4
**Histology**									
Ductal	5909	472	8.0	3912	66.2	1286	21.8	239	4.0
Lobular	567	45	7.9	361	63.7	140	24.7	21	3.7
Other	496	71	14.3	335	67.5	81	16.3	9	1.8
**Tumour Size (mm)**									
<20	4847	445	9.2	3452	71.2	859	17.7	91	1.9
≥20–≤50	2125	143	6.7	1156	54.4	648	30.5	178	8.4
**Positive Lymph Node**									
0	5453	498	9.1	3717	68.2	1169	21.4	69	1.3
1–3	1295	85	6.6	779	60.2	323	24.9	108	8.3
4+	224	5	2.2	112	50.0	15	6.7	92	41.1
**Lympho-vascular Invasion**									
Yes	5799	508	8.8	3885	67.0	1254	21.6	152	2.6
No	1173	80	6.8	723	61.6	253	21.6	117	10.0
SYSTEMIC TREATMENT VARIABLES									
**Systemic Treatment**									
None	2303	275	11.9	1464	63.6	545	23.7	19	0.8
Both	932	44	4.7	602	64.6	180	19.3	106	11.4
Chemotherapy	711	47	6.6	449	63.2	141	19.8	74	10.4
Hormonal therapy	2961	204	6.9	2064	69.7	624	21.1	69	2.3
Unknown	65	18	27.7	29	44.6	17	26.2	1	1.5

^1^ Breast Conserving Surgery (BCS); ^2^ Breast Conserving Surgery plus Radiotherapy (BCS + RT); ^3^ Mastectomy (MTX); ^4^ Mastectomy plus Radiotherapy (MTX + RT); ^5^ Interquartile Range; ^6^ Oestrogen Receptor (ER); ^7^ Progesterone Receptor (PR).
